# Investigation of Indazole Unbinding Pathways in CYP2E1 by Molecular Dynamics Simulations

**DOI:** 10.1371/journal.pone.0033500

**Published:** 2012-03-19

**Authors:** Zhonghua Shen, Feixiong Cheng, You Xu, Jing Fu, Wen Xiao, Jie Shen, Guixia Liu, Weihua Li, Yun Tang

**Affiliations:** Shanghai Key Laboratory of New Drug Design, School of Pharmacy, East China University of Science and Technology, Shanghai, China; Universität Erlangen-Nürnberg, Germany

## Abstract

Human microsomal cytochrome P450 2E1 (CYP2E1) can oxidize not only low molecular weight xenobiotic compounds such as ethanol, but also many endogenous fatty acids. The crystal structure of CYP2E1 in complex with indazole reveals that the active site is deeply buried into the protein center. Thus, the unbinding pathways and associated unbinding mechanisms remain elusive. In this study, random acceleration molecular dynamics simulations combined with steered molecular dynamics and potential of mean force calculations were performed to identify the possible unbinding pathways in CYP2E1. The results show that channel 2c and 2a are most likely the unbinding channels of CYP2E1. The former channel is located between helices G and I and the B-C loop, and the latter resides between the region formed by the F-G loop, the B-C loop and the β1 sheet. Phe298 and Phe478 act as the gate keeper during indazole unbinding along channel 2c and 2a, respectively. Previous site-directed mutagenesis experiments also supported these findings.

## Introduction

The cytochromes P450 (CYPs) are a superfamily of heme-containing monooxygenases that catalyze metabolism and activation of a variety of endogenous and exogenous compounds. Catalyzing biosynthesis and degradation of chemicals is the most common function of P450s in microorganisms, plants and animals. Thus, CYPs are absolutely indispensable from the biological and pharmaceutical viewpoints [1].

To date, crystal structures of more than thirty CYPs from different species have been determined. Despite the low sequence identity (∼20%) between some CYPs, their structures display a highly similar structural fold. The active site of CYPs contains a heme group and the iron atom is tethered to CYP via a highly conserved cysteine. Most CYPs adopt a closed conformation, in which the active site is deeply buried in the protein core and inaccessible to the protein exterior. Thus, the question of how ligand enters/exits the active site to undergo oxidation or inhibition has drawn much attention.

Random expulsion molecular dynamics and steered molecular dynamics have been used to investigate the possible channels in the bacterial CYPs: CYP101, CYP102, and CYP107A [2], [3]. A channel named channel 2a (the nomenclature is from Wade's work [4]) was found common in these bacterial CYPs. However, the channel opening mechanisms are different in these CYPs [5]. In contrast to soluble bacterial CYPs, mammalian CYPs are membrane-anchor proteins through the N-terminal trans-membrane helix, so the pathways for ligands passage may differ. Wade's group found pw2c to be the predominant pathway in mammalian CYP2C5 [6] and they subsequently systematically defined the channel classes [4] according to the geometric analysis with CAVER, a program designed for channel identification in macromolecules [7]. By using molecular dynamic simulations, we previously analyzed in detail the ligand unbinding process in mammalian CYP3A4, CYP2B1 and CYP2A6 [8], [9], [10]. Two pathways, pw2c and pw2e, were found to be involved in ligand passage in these CYPs. Recently, the substrate access/egress routes in CYP2C9 were investigated by different groups [11], [12]. The results indicated that CYP2C9 preferred pw2 (pw2a, 2b, 2c, and 2e) and the solvent channel for substrate access or product release. In addition, the shape and oxidized position of the large products would determine the exit channel due to alterations of rotational and translational freedom of the product in the active site [13]. These findings point out that though CYPs have the highly structural similarity, there exist different channels for ligands passage and the opening mechanisms differ in different CYP isoforms.

CYP2E1, a member of CYP superfamily in human liver microsome, catalyzes oxidative reaction of many low molecular weight xenobiotics and engogenous fatty acids. For example, ethanol can be converted to acetaldehyde and acetate by CYP2E1 [14]. The size of the active site in CYP2E1 is the smallest among human CYP crystal structures available [15]. This characteristic might explain why CYP2E1 is more advantageous to metabolize small weight compounds, and at the same time creates a question whether the pathways in CYP2E will differ from those found in other mammalian CYPs. The distinct substrate binding in CYP2E1 active site has led to a speculation that the rotation of Phe478 might allow the connection of the active site and the adjacent cavity to form the access channel [15]. However, such a mechanism needs to be validated by further evidence.

In this study, we first explored the potential openings in CYP2E1 crystal structures using the MOLE program [16]. Then, random acceleration molecular dynamics (RAMD) simulations [3] and steered molecular dynamics (SMD) simulations [2] were performed to identify the possible channels and characterize the dynamic process during the ligand unbinding. Finally, potential of mean force (PMF) was computed to compare the ligand pathway selectivity. This study aims to find not only the effects of the key residues lining the channel on ligand unbinding, but also the associated unbinding mechanisms.

## Materials and Methods

### Identification of channels in CYP2E1 crystal structures

**Figure 1:**
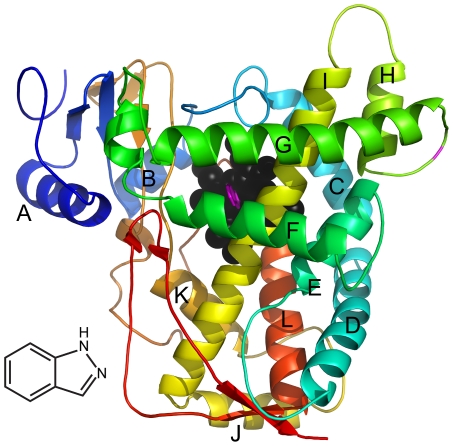
The crystal structure of CYP2E1 complexed with indazole. The major helices are labeled. Heme is shown as black spheres; indazole is shown as magenta sticks. All figures were generated with Pymol.

Till date, five CYP2E1 crystal structures have been solved and deposited in Protein Datebase Bank (PDB), including three fatty acids-bound structures and two small weight inhibitor-bound structures. In this study, the structure of CYP2E1 in complex with indazole (PDB code 3E6I, at 2.2 Å resolution) was chosen (Figure 1). In order to explore potential access/egress channels in the static crystal structure, the MOLE program [16], a plugin in Pymol, was used. The CYP2E1 crystal structure was first loaded in Pymol, and then the Fe atom in heme was set as the starting point and the number of tunnels was set to twenty.

### Molecular dynamics (MD) simulation

The protonation states of ionizable residues were determined by PROPKA [17], which can predict the p*Ka* values of protein residues. Based on the calculated p*Ka* values, His107, His152, His188, His226, His232, His253, His254, His278 and His326 were assigned to be fully protonated at both nitrogens. His109, His355, His370 and His475 were protonated at δ nitrogen and other histidines at ε nitrogen. Glu477 was in the protonated state.

The initial model of indazole was extracted from the crystal structure of the CYP2E1-indazole complex (PDB code 3E6I). Structural optimization of indazole was conducted at the B3LYP/6-31G** level using Gaussian03 and the restrained electrostatic potential (RESP) fitting procedure was used for charge derivation based on the optimal conformation. The force field parameters of indazole were supplied by AMBER10. The LEaP program of AMBER10 produced force field, topology and coordinate information for the following simulations. The protein was solvated in a truncated octahedron periodic box of 10 Å buffer of TIP3P water. Chlorine counter ions were added to neutralize the system.

The MD simulations were performed using Amber10. Energy minimization was carried out with a decreasing harmonic force constraint on the protein. The minimized system was gradually heated from 0 K to 300 K under the NVT ensemble condition and equilibrated for 50 ps at 300 K, followed by 10 ns of MD simulation under the NPT ensemble condition. The SHAKE algorithm was adopted for bond length constraints. The time step and nonbonding interaction cutoff radius were set to 2 fs and 10 Å, respectively.

### Random acceleration molecular dynamics (RAMD) simulation

In contrast to MD, RAMD simulation applies an artificial force on the center of mass of indazole, which would expulse the ligand from the active site to the protein surface. The initial direction of the force is chosen randomly. When the ligand moves less than the minimum distance (r_min_) within the fixed time (N×Δt, N is the number of steps, Δt is the time step), the direction of the force would change, such that the ligand should meet an average velocity during the limited time (N×Δt). The advantage of this method is that RAMD simulation does not require a pre-determined direction compared to steered molecular dynamics (SMD). In this mode, RAMD can identify egress routes for the ligand with the force constant as low as possible.

Two magnitudes of accelerations, 0.15 and 0.2 kcal•Å^−1^•g^−1^, were used in the simulations. The N was set to 40 steps. The r_min_ was set to 0.005, 0.01, and 0.02 Å, separately. To ensure the reliability of the experiment, three snapshots extracted from the MD simulation were chosen as the starting structures for RAMD. 45 trajectories were generated for each snapshot and a total of 135 RAMD simulations were performed for three snapshots. The time cutoff for each RAMD simulation was set to 3 ns. If the ligand could not successfully exit within 3 ns of RAMD simulations, the trajectory was abandoned. The numbers of the successful egress trajectories are summarized in Table 1.

**Table 1:** Statistical summary of RAMD simulations of CYP2E1.

Channel	A (kcal•Å^−1^•g^−1^)	r_min_ (Å)	N (steps)	Trajectory length (ps)	No. of successful egress trajectories		
					7 ns	8 ns	9 ns
2c	0.15, 0.2	0.01, 0.02, 0.005	40.	605–1708	9	8	12
2a	0.15, 0.2	0.01, 0.02, 0.005	40	558–1723	8	10	7
2b	0.2	0.01, 0.02, 0.005	40	641–1727	3	2	3
2f	0.2	0.01, 0.02	40	1008–3311	2	4	1
3	0.2	0.01, 0.02	40	1282–3274	3	0	1
Solvent	0.2	0.01, 0.02	40	1340–1499	2	2	3
Water	0.2	0.01, 0.02	40	1906–3112	1	3	2
1	0.2	0.02	40	2262–2709	1	1	1
2d	0.2	0.02	40	2534–2893	1	1	0

### Steered molecular dynamics (SMD) simulation

SMD applies an external force on the center of mass of the ligand to pull it out in a determined direction. The directions towards the outside protein were determined from the statistical results of RAMD trajectories. The pulling direction was set by defining two groups, the original location of indazole in the active site and the C_α_ atom of Gln327 and Pro145, which pointed to the channel 2c and 2a, respectively. The constant velocity SMD was used in this simulation. The spring constant and the velocity were set to 4 kcal•mol^−1^•Å^−2^ and 0.01 Å•ps^−1^, respectively. In order to avoid the large displacement of structure, positional restraints of two C_α_ atoms of Lys288 and Met317 in helix I and the Fe atom were applied by using a harmonic potential with a force constant of 50 kcal•mol^−1^•Å^−2^. 30 SMD trajectories using different random seeds were generated repeatedly for each channel. The displacement distance of indazole and the simulation duration were set to 30 Å and 3 ns, respectively. From these simulations, the statistics about the force and work was obtained.

The force imposed on the ligand is defined as:
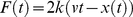
(1)where 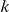 is the force constant; 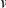 is the velocity; and 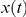 is the location of the ligand at 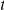 time.

The work can be calculated as follow:
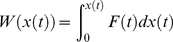
(2)


### Potential of mean force (PMF)

The values of work obtained from SMD simulations were used to compute free energy through Jarzynski's equality [18], according to the following equation.
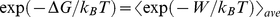
(3)


The equality means that equilibrium free energies can be derived from non-equilibrium calculation through the work between two states and averaging over the starting state. Sufficient simulations are required to accurately estimate the free energies. However, in reality, only a limited number of trajectories can be sampled. Thus, many methods were developed to reduce the statistical error. In this work, the cumulative integral (CI) extrapolation method developed by Ytreberg and Zuckerman was used [19], which has been proved to be accurate by reducing the required data with 5–40 fold and obtains satisfactory results in practical cases [19], [20]. A 25 ps (0.5 Å) sampling window was used in order to obtain converged PMF data. The uncertainty was estimated using the bootstrap method according to the previous studies [8], [21].

## Results and Discussion

### Exploration of channels in CYP2E1 crystal structures

Five crystal structures of CYP2E1 including small weight inhibitors and fatty acids bound complexes are available in PDB. In these static CYP2E1 crystal structures, several potential channels have been detected by the MOLE program (Figure S1). However, the results show that the bottleneck radii of all the acquired channels are smaller than 1.4 Å, which is the radius of the water molecule (Table S1). This implies that the active site of CYP2E1 is inaccessible to the protein exterior in their crystal structures. Compared with CYP2E1, the crystal structure of CYP2B4 has a large cleft that can conveniently allow the ligand to enter the active site without considerable constrictions [22]. The major influencing factor in the formation of channels in CYPs was considered to be the movement of the B-C loop, the F and G helices and the F-G loop, which were proved to be more flexible [23]. Therefore, dynamic simulations were adopted to further examine the shift of the secondary structures and the action mechanisms in details.

### RAMD simulations

**Figure 2:**
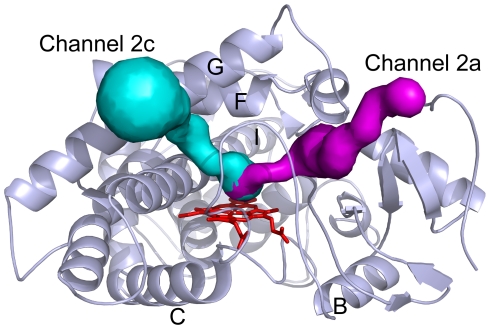
Indazole unbinding routes from CYP2E1 illustrated by using the MOLE program. Channel 2c is shown in cyan and channel 2a is shown in magenta. The major secondary elements of CYP2E1 are labeled.

Since no obvious channel is open enough for ligands to pass through in the CYP2E1 crystal structures, an effective method RAMD was used to identify the possible channel. RAMD simulations exert a random external force on the ligand to accelerate ligand binding/unbinding. Prior to RAMD simulations, 10 ns conventional MD simulations were performed in order to obtain an equilibrium system. The results showed that total energy and heavy atom root-mean-square deviation (RMSD) were convergent and the system was in a stable state after 4 ns (**Figure** S2). In order to improve the credibility of simulations, three snapshots at 7, 8 and 9 ns were used as the initial structures for RAMD simulations. The trajectory information is summarized in **Table 1**, which reflects that the influence of starting structures on the channel selection is not notable. Two channels, 2a and 2c, have higher occurrence frequencies than other channels (Figure 2).

There are nine channels in CYP2E1 identified by RAMD simulations, which are 2a, 2b, 2c, 2f, 2d, 1, 3, solvent and water channel. Channel 2 has the subclasses 2a, 2b, 2c 2d and 2f. These channels were also found in many other CYPs, such as CYP3A4 [9], [13], 2C5 [6], 2B1 [10], 2A6 [8], 101, and 102A1 [2], [3]. Channel 2a is located between the F-G loop, the B-C loop and the β1 sheet. Channel 2b is located between the B-B′ loop, β1 and β3 sheets. Channel 2c is located between G, I helixes and B-C loop. Channel 2d is located between N terminus and helix A. Channel 2f is located between helix F′ and the β5 sheet. Channel 1 is located between C and H helices. Channel 3 is located between F and G helixes. Solvent channel is located between E, F, and I helices. Water channel is at the lower part of the B-C loop.

From the statistical data in **Table 1**, different frequencies were observed for nine channels. The ratios of channel 2a and channel 2c are about 27% and 32%, respectively. The remaining seven channels were observed rarely. Moreover, the smaller acceleration, 0.15 kcal•Å^−1^•g^−1^, can lead to ligand unbinding only along channel 2a and 2c. In light of the principle of RAMD and many studies conducted previously [3], [6], [8], channel 2a and 2c will be most likely as the ligand channels of CYP2E1. However, preference between these two channels serving as the unbinding pathway needs further investigation.

### SMD simulations

**Figure 3:**
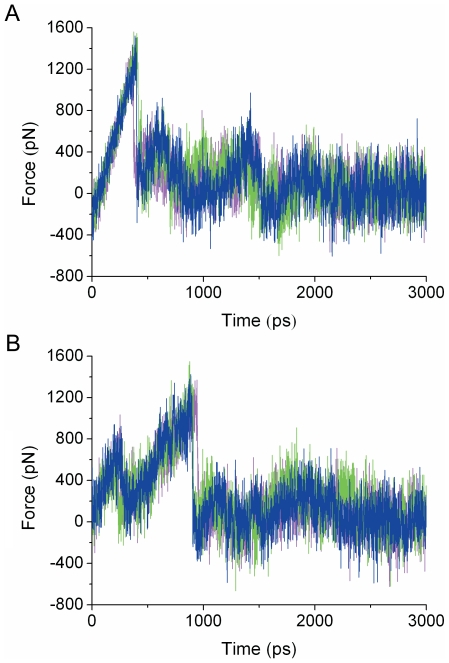
Force profiles along channel 2c (A) and 2a (B). Three different lines represent data from three representative SMD simulations.

Compared to RAMD, SMD is suitable to estimate expulsion force and energy barriers. Each SMD trajectory lasted for 3 ns, and thirty trajectories were produced repeatedly for each channel. **Figure 3** show the force profiles during indazole unbinding along channels 2a and 2c (also see Figure S3). The results show that the largest forces along channel 2c and 2a are almost identical. However, the simulation time needed to reach the highest force differs. Channel 2c required a stable increasing force in the initial process of unbinding, whereas channel 2a had a force that fluctuated greatly from the beginning to 950 ps.

### Unbinding along channel 2c

**Figure 4:**
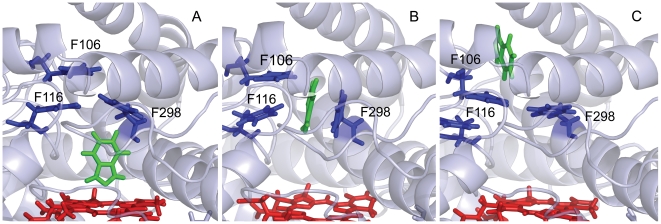
Snapshots of the relative positions of indazole and the protein throughout the SMD simulation along channel 2c (A, 200 ps; B, 500 ps; and C, 800 ps). Indazole is shown as green sticks. Heme is shown as a red stick. Phe106, Phe116 and Phe207 are shown as blue sticks.

From 0 ps to 390 ps, the force profile is in the first stage. The inhibitor stayed in the active site above the heme group and the force sharply increased to the largest value. The nitrogen atom of indazole coordinated with the heme iron and the nearby nitrogen atom had the hydrogen bonding with Thr303, which was considered to be a conserved residue and played important role in CYP2E1 activity. Leu337, Ile85 and Ala268 held the inhibitor in the active site through hydrophobic contacts. More importantly, the Phe-cluster including Phe106, Phe116, Phe207, Phe298, and Phe478, formed a ceiling on the binding pocket and blocked the channel 2c, as shown in Figure 4A. Especially, Phe298 imposed ultimate restrictions on the inhibitor, and then the applied force reached the peak.

**Figure 5:**
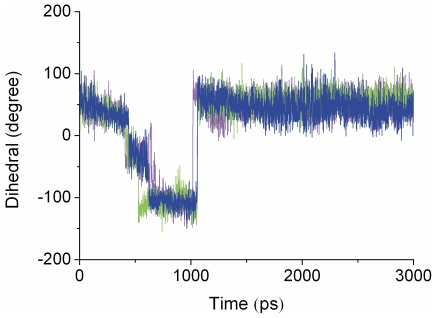
Variation of the benzene ring of F298 is shown as a function of time during SMD simulation. Three different lines represent data from three representative SMD simulations.

In the second stage from 390 ps to 2000 ps, because indazole broke through the obstruction caused by Phe-cluster, the force decreased obviously. At 390 ps, the benzene rings of Phe116 and Phe298 rotated due to the contacts with indazole. These actions open up the channel 2c and indazole began to leave the binding site along the channel with a relatively rapid speed. From 400 ps to 900 ps, indazole formed hydrophobic interactions with Ile85 and Ala268 and a hydrogen bond with the carbonyl group of Asp264. As indazole moved towards the entrance of channel 2c slowly, the π-stacking interaction between indazole and Phe298 got disrupted and the benzene ring of Phe298 rotated nearly 360 degrees as a revolving door (**Figure** 4B, 4C and 5). From 900 ps to 1100 ps, the hydrogen bond between Asp264 and indazole was broken and the π-stacking interactions disappeared, the force decreased conspicuously in agreement with this process. At the entrance of channel 2c, indazole formed the hydrogen bond with His109 and the hydrophobic interactions with Val239, Lys243, Val291 and Ala294.

In the last stage, the hydrogen-bonding and the hydrophobic interactions between the inhibitor and CYP2E1 were destroyed, which indicates that indazole has moved out of the channel completely. The force profile was only influenced by interactions between the solvent molecules and indazole.

### Unbinding along channel 2a

In the first 250 ps, the force profile increased gradually when indazole was held in the binding site by the non-bonded interactions, involving a hydrogen bond with Thr303, a coordinate bond with the heme iron and the hydrophobic contacts with Ile85, Ala286, Glu271 and Leu337. The force went up to the first peak at 210 ps. These effects are similar to those along channel 2c. The breakages of these interactions are considered to be the first step of indazole unbinding.

**Figure 6:**
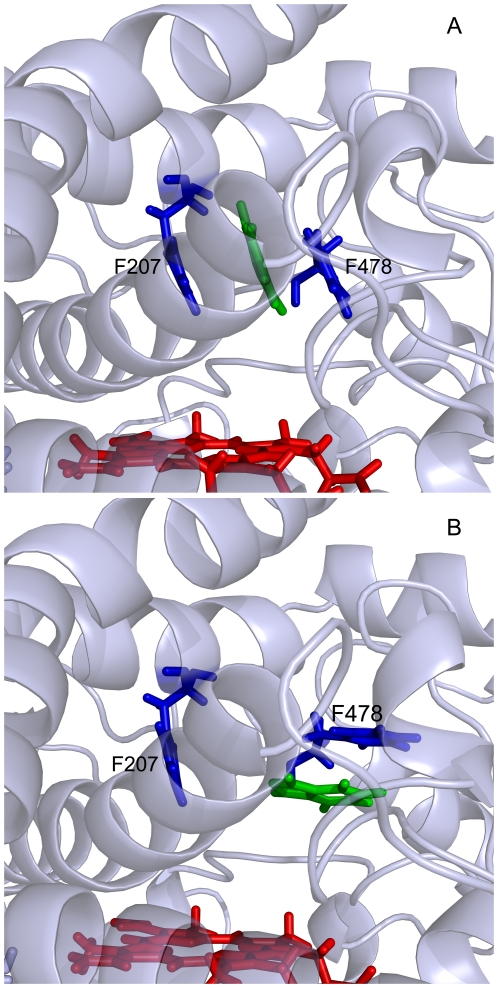
Snapshots of the relative positions of indazole and the protein throughout the simulation along channel 2a (A, 490 ps; B, 980 ps). Indazole is shown as green sticks. The heme is shown as a red stick. Phe207 and Phe478 are shown as blue sticks.

During the period of 370 ps to 950 ps, the highest peak of force profile emerged, which was mainly caused by the influence of the Phe-cluster. With the rotation of the benzene rings of Phe207 and Phe478, the benzene rings form π-π interactions with indazole just like a sandwich (**Figure** 6A). Indazole was held between Phe478 and Phe207 above the active site temporarily. The rotation of the benzene ring of Phe478 blocked the channel 2a to prevent indazole from moving away.

After 950 ps, the single-bond between C2 and C3 in the side chain of Phe478 began to revolve and the π-π interactions were destroyed rapidly. When the benzene ring of Phe478 was parallel to the direction of the channel 2a (Figure 6B), indazole moved out fast. At this point, channel 2a connects the solvent to the buried center of CYP2E1. The force profile decreased sharply from the peak at 950 ps to the local minimum at 960 ps. After indazole leaving from the active site completely, the benzene ring of Phe478 returned to its original position.

At the entrance of channel 2a, indazole had hydrophobic interactions with Asn219, Gln75 and Met77 with the force increasing slightly at about 2150 ps. After 2200 ps, indazole left the protein thoroughly.

### PMF analysis

**Figure 7:**
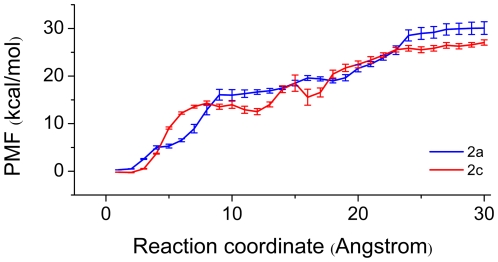
PMF profiles along channel 2a (magenta) and channel 2c (green). The error bar indicates the standard deviation of PMF by the bootstrap approach.

To further compare the preference of channel 2c and 2c serving as indazole unbinding channel, PMF was reconstructed to estimate the free energy change during the inhibitor unbinding. The PMF profiles in **Figure 7** show the energy changes in the course of indazole dissociation along channel 2c and 2a.

The PMF along channel 2c increased sharply in the first 6 Å displacement mainly due to the resistance of Phe298 located in the active site. Then the PMF increased again because of the formation of hydrogen bond between His109 and indazole after 16 Å displacement. After 23 Å in the reaction coordinate, the PMF was converged. Compared to channel 2c, the PMF along channel 2a increased gently at first. The benzene rings of Phe207 and Phe478 rotated more significantly to influence indazole unbinding. After 15 Å displacement, the PMF increased gradually due to the movement of indazole, which resulted in a larger displacement of Phe478 and the strong hydrophobic contacts in channel 2a.

Although the PMF profile of indazole unbinding along channel 2c slightly differed from that along channel 2a, both the PMF values converged to nearly 30 kcal•mol^−1^.

### Both channel 2c and 2a serve as indazole unbinding pathways

In light of the results of the RAMD and SMD simulations, which show that these two channels have a large proportion of all channels, the similar maximum values of the force and the converged PMFs, we conclude that both channel 2c and 2a can be used as the inhibitor unbinding channels in CYP2E1. Previous researches have shown that there are multiple channels in some CYPs, such as CYP2B1 [10] and CYP2C9 [13].

Channel 2c has previously been identified to be a channel in several CYPs, such as CYP2C5, 2A6 and 2B1. In CYP2C5, the formation of channel 2c depended on breakage of the hydrogen bond between K241 and V106, since this action can improve the flexibility of the B′ helix and B-C loop lining channel 2c [6]. In CYP2A6, the small force and energy barriers allowed channel 2c to be the most likely channel for substrate egress. In CYP2B1, channel 2c was considered to be the one of the most preferred channels for substrate exiting the active site [10]. In this study, when indazole moved out of the protein, the contacts between Phe-cluster and the inhibitor should be overcome. This is similar to CYP2A6 [8]. In addition, the mutant H109F, which is located in the channel, lowers the activity and stability of the enzyme [24]. Moreover, B′ helix was also proved to be quite important in the formation of channel 2c. These lines of evidence show that channel 2c was likely to serve as the channel in CYP2E1.

Channel 2a was confirmed as the major channel in prokaryotic CYPs such as CYP101, in which the unbinding work and force for camphor along channel 2a were lower than other channels through RAMD simulations and only small backbone motions were required for camphor leaving. Mammalian CYPs are anchored to the ER-membrane through trans-membrane N-terminus. Recently, the membrane-bound CYP2C9 model was constructed, and the membrane facilitated the opening of channel 2a [12]. The lipophilic substrates could pass through the membrane into channel 2a to participate in metabolism [6], In addition, the mutant R76H, which is located near channel 2a, has only 36% of the catalytic activity in hydroxylation of chlorzoxazone compared to CYP2E1 wild type [25], suggesting an important function of Arg76 in enzymatic activity. Taken together, channel 2a has high possibility to serve as one of ligand channels in CYP2E1.

### Gating mechanisms

Our results demonstrate that channels were influenced by the different gating mechanisms and the level of difficulty in inhibitor dissociation depended on the actions of the gating residues. Although CYPs have similar fold, many studies illustrated that different CYPs have different channels and gating mechanisms. Gating residues may have important roles in the activities of CYP.

In channel 2c, the Phe-cluster including F106, F116, F207, F298 and F478 serves as the ceiling above the active site and prevented the channel from connecting the active site to the outside of the protein. This phenomenon was also found in CYP2A6 and CYP2B1. When the ligand passed through this cluster, both force and energy reached the maximum value with the movement of Phe-cluster. Moreover, the ring of F298 rotated nearly 360 degrees and had the π-π interaction with indazole that further hindered the unbinding process of product. Therefore, the F298 was considered to act as the gate keeper in channel 2c.

In channel 2a, Phe478 was considered to be the gate-keeper in the whole process due to its key position which needs largest force. The appropriate swing of the ring of Phe478 made the connection between the active site and the nearby channel to the surface of the protein, which was also observed in CYP101. Phe478 performed as a regulator to control the switch of channel 2a. In addition, the mutant F478V resulted in an increase in 7-ethoxy-4-trifluoromethylcoumarin deethylation activity but a decrease in *p*-nitrophenol hydroxylation activity than CYP2E1 wild type, indicating that this residue is very important in metabolic reactions [26]. To sum up, the phenylalanine gating mechanisms play a key role in the unbinding process in CYP2E1, though the gate-keeper residues differ in channel 2a and 2c.

### Conclusions

CYP2E1 is a member of CYP superfamily, characterized by its catalysis toward small weight compounds and large fatty acids. Though the fold structure is similar to other P450s, the active site of CYP2E1 is the smallest in the available human CYP crystal structures. From the solved crystal structure, no obvious channel is open enough to permit ligand passage, which was analyzed by the MOLE program. Therefore, MD simulations were applied to explore the possible channels. RAMD simulations show that channel 2c and 2a have about 59 percent of the successful egress. SMD were then performed to calculate the force and energies in the inhibitor unbinding process. Finally, PMF was calculated to compare the unbinding free energies. The results show that channel 2a and 2c have the similar maximum values of the forces and the PMFs, which are mostly likely to be the dominant channels of CYP2E1 compared with other channels. Some residues lining channel 2a and 2c were identified to be important for the inhibitor unbinding, which have been supported by previous site-directed mutagenesis. Our results will be helpful in understanding the channel selectivity of P450s and the unbinding mechanism of the inhibitor in CYP2E1.

## Supporting Information

Figure S1 **The major egress channels from the active site of indazole-bound CYP2E1 complex, identified by the MOLE program.** The channels share the slate color. Heme is shown as a red stick. The major secondary elements of CYP2E1 are labeled.
(TIF)

Figure S2 **Total energy (A) and RMSD (B) are shown as a function of time during the molecular dynamic simulation.**
(TIF)

Figure S3 **The variation of time and force with respect to reaction coordinate in channel 2c (A and B) and channel 2a (C and D).** Three different lines represent data from three representative SMD simulations.
(TIF)

Table S1 **Minimum bottleneck values for substrate channels observed by MOLE.**
(DOC)
