# Correction: Investigation of Indazole Unbinding Pathways in CYP2E1 by Molecular Dynamics Simulations

**DOI:** 10.1371/annotation/86d9f6df-7175-467c-a9ff-94eac53af128

**Published:** 2012-09-05

**Authors:** Zhonghua Shen, Feixiong Cheng, You Xu, Jing Fu, Wen Xiao, Jie Shen, Guixia Liu, Weihua Li, Yun Tang

There is an error in the Funding section. The Funding section should read: Shanghai Natural Science Foundation (Grant No. 10ZR1407000), Fundamental Research Funds for the Central Universities, the Scientific Research Foundation for the Returned Overseas Chinese Scholars, State Education Ministry, the Program for New Century Excellent Talents in University (Grant No. NCET-08-0774), and the Shanghai Committee of Science and Technology (Grant 11DZ2260600). The funders had no role in study design, data collection and analysis, decision to publish, or preparation of the manuscript. 

