# Supplementary figures and images for: Investigation of Indazole Unbinding Pathways in CYP2E1 by Molecular Dynamics Simulations
Source: PLoS One. 2012 Mar 19;7(3):e33500. doi: 10.1371/journal.pone.0033500 (PMC3307744; doi:10.1371/journal.pone.0033500)

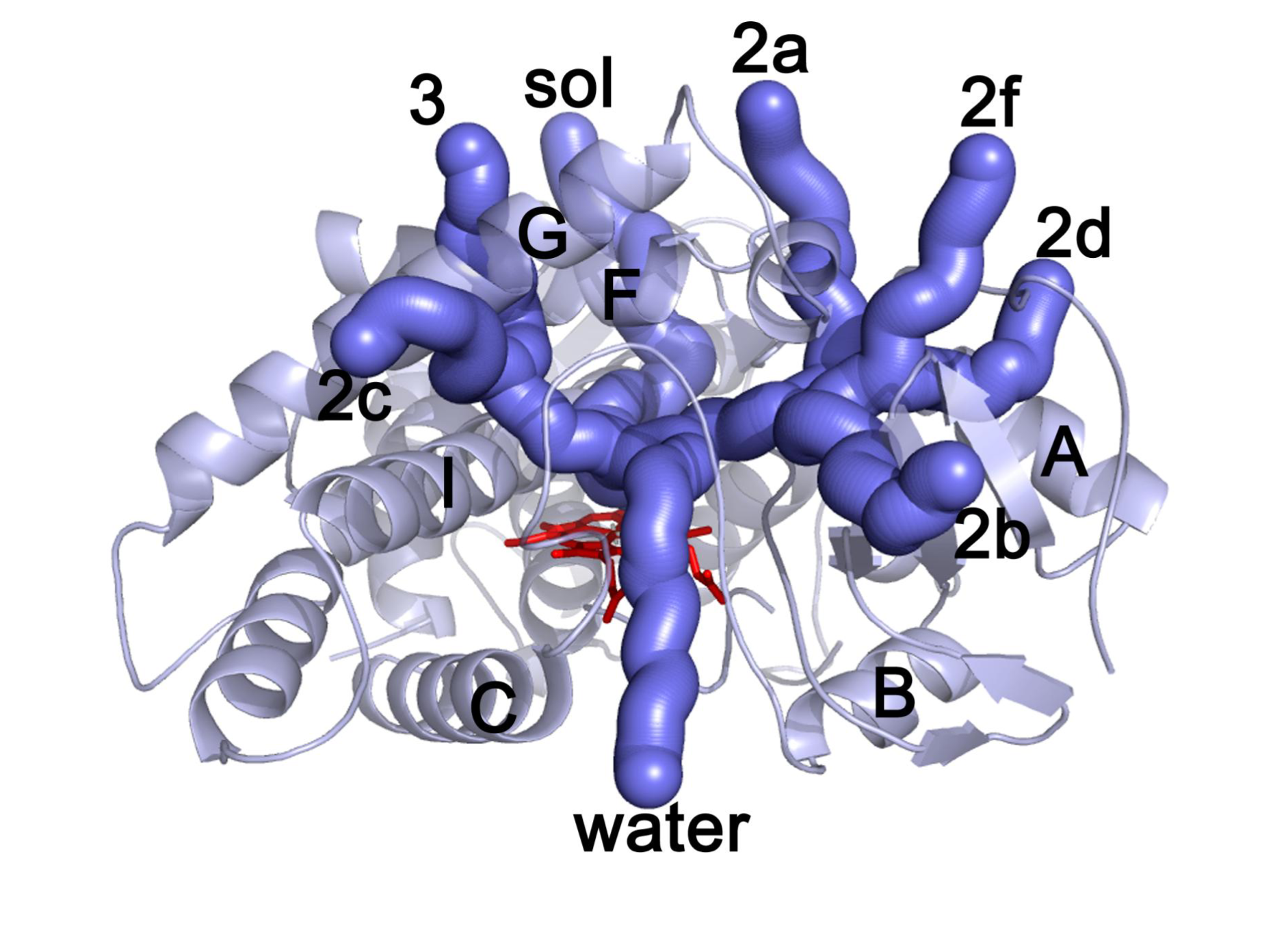

Supplement: Figure S1 — The major egress channels from the active site of indazole-bound CYP2E1 complex, identified by the MOLE program. The channels share the slate color. Heme is shown as a red stick. The major secondary elements of CYP2E1 are labeled. (TIF) [file pone.0033500.s001.tif]

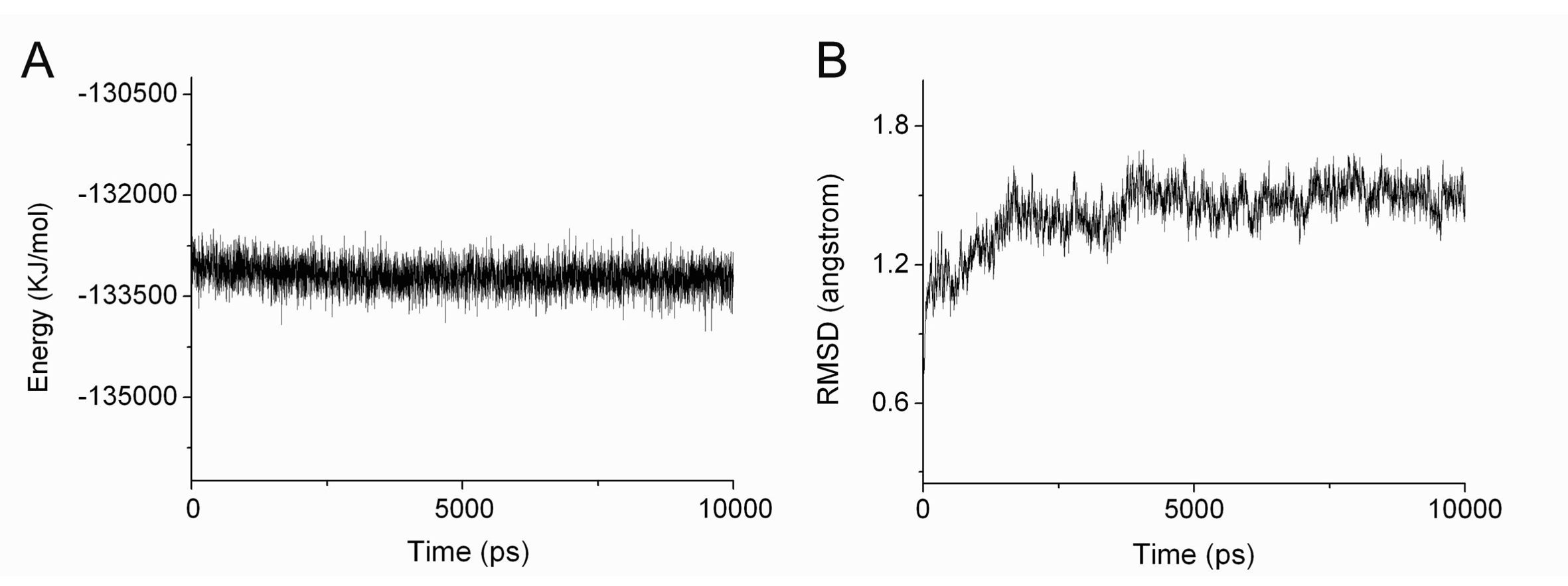

Supplement: Figure S2 — Total energy (A) and RMSD (B) are shown as a function of time during the molecular dynamic simulation. (TIF) [file pone.0033500.s002.tif]

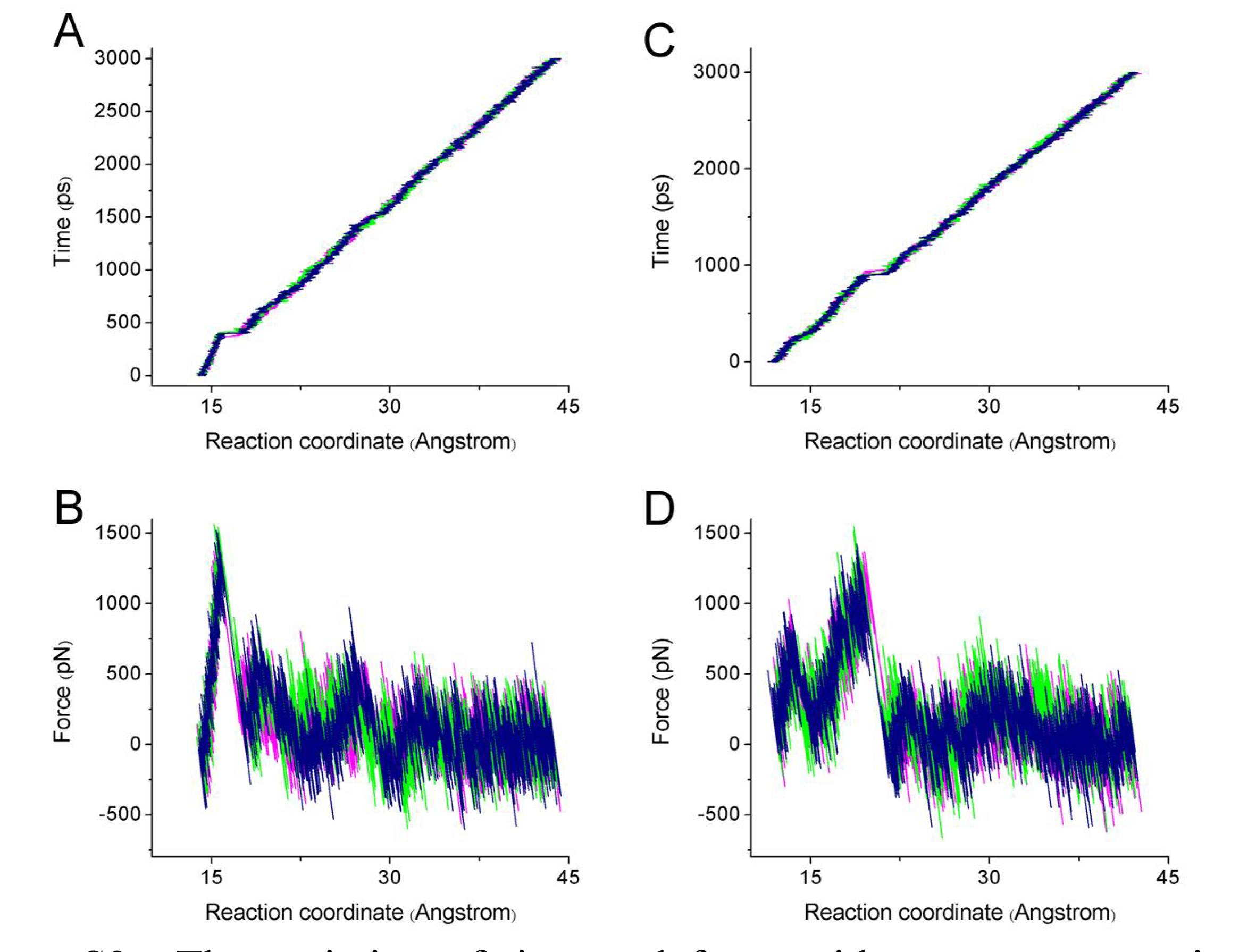

Supplement: Figure S3 — The variation of time and force with respect to reaction coordinate in channel 2c (A and B) and channel 2a (C and D). Three different lines represent data from three representative SMD simulations. (TIF) [file pone.0033500.s003.tif]
