# Supplementary material for: Investigation of Indazole Unbinding Pathways in CYP2E1 by Molecular Dynamics Simulations
Source: PLoS One. 2012 Mar 19;7(3):e33500. doi: 10.1371/journal.pone.0033500 (PMC3307744; doi:10.1371/journal.pone.0033500)
Supplement: Table S1 — Minimum bottleneck values for substrate channels observed by MOLE. (DOC) [file pone.0033500.s004.doc]

Table S1

| PDB_ID_chain | Bottleneck radii values for channels observed by MOLE (Å) | | | | | | | | |
| --- | --- | --- | --- | --- | --- | --- | --- | --- | --- |
|  | 1 | 2a | 2b | 2c | 2e | 2f | 3 | S | W |
| 3E4E_A | 0.55 | 0.75 | 0.89 | 0.74 |  | 0.72 | 0.74 | 0.66 |  |
| 3E4E _B | 0.63 | 0.78 | 0.96 | 0.88 |  | 0.89 | 0.64 | 0.59 |  |
| 3E6I _A | 0.61 | 1.04 |  | 0.56 |  | 0.73 | 0.43 | 0.62 | 0.62 |
| 3E6I _B | 0.55 | 0.80 | 1.06 | 0.85 | 0.77 | 0.70 | 0.64 |  |  |
| 3LC4_A | 0.86 | 0.95 | 1.26 | 1.00 |  | 0.59 | 0.73 | 0.59 |  |
| 3LC4_B |  | 0.86 | 1.22 | 0.94 |  | 0.64 | 0.75 |  |  |
| 3KOH_A |  | 0.61 | 1.33 | 0.70 |  | 0.77 | 0.76 | 0.79 |  |
| 3KOH _B | 0.49 | 0.64 | 1.10 |  |  | 0.91 | 0.74 | 0.67 |  |
| 3GPH_A | 0.47 | 1.11 | 1.21 | 0.72 |  | 0.81 | 0.57 | 0.63 |  |
| 3GPH _B | 0.56 | 1.02 | 0.81 |  |  | 0.66 | 0.52 | 0.67 |  |
